# Relationship between internet addiction and academic performance of undergraduate medical students of Azad Kashmir

**DOI:** 10.12669/pjms.36.2.1061

**Published:** 2020

**Authors:** Arslaan Javaeed, Raheema Jeelani, Shazia Gulab, Sanniya Khan Ghauri

**Affiliations:** 1 Dr. Arslaan Javaeed, MBBS, M.Phil., MHPE. Poonch Medical College, Rawalakot, Azad Kashmir, Pakistan; 2 Dr. Raheema Jeelani, MBBS. Poonch Medical College, Rawalakot, Azad Kashmir, Pakistan; 3 Dr. Shazia Gulab, MBBS. Poonch Medical College, Rawalakot, Azad Kashmir, Pakistan; 4 Dr. Sanniya Khan Ghauri, MBBS, MRCEM. Department of Emergency Medicine, Shifa International Hospital, Islamabad, Pakistan

**Keywords:** Academic performance, Azad Kashmir, Internet addiction, Medical students, KAP study

## Abstract

**Objective:**

To assess the relationship between internet addiction (IA) and academic performance among the medical students of Azad Kashmir, Pakistan.

**Methods:**

A cross-sectional study was done involving 316 medical students of Poonch Medical College, Azad Kashmir, Pakistan from May 2018 to November 2018. Dr. Young’s Internet Addiction Test questionnaire was used as the tool of data collection. The questionnaire contained twenty 5-points Likert scale questions to assess internet addiction. IA score was calculated and the association between IA and academic performance was observed by Spearman Rank Correlation test. Relationship between baseline characteristics of the medical students and IA was also seen.

**Results:**

Eighty-nine (28.2%) medical students fell under the category of ‘severe addiction’ and most importantly only 3 (0.9%) were not internet addicted according to Dr. Young’s questionnaire. Internet addicted medical students scored significantly poor in their exams (p. <.001). One hundred thirty one (41.4%) students with a median IA score of 45 scored in the range of 61-70% marks as compared to 3 (0.9%) students with a median IA score of 5, secured greater than 80% marks.

**Conclusion:**

This study and many other previous studies have revealed that internet addiction affects academic performance. The number of internet users is ever increasing therefore, the number of internet misusers will also increase. If no step is taken to control internet addiction, it may cause a serious impact in the future.

## INTRODUCTION

The beginning of the 21^st^ century has witnessed an explosive growth of internet usage worldwide, particularly in developing countries like Pakistan.^1^ Due to the advanced development of network construction in universities, the number of Internet-using university students is increasing.^2^ Medical students have an enormous opportunity to keep them always updated with the exponential growth of knowledge because of potential progression of Internet throughout the world that enables them to become a lifelong learner.^3^ The term Internet Addiction Disorder was first proposed by Ivan Goldberg for pathological, compulsive Internet usage.^4^ The criteria for this disorder are based on similar criteria for substance abuse disorders in the DSM-IV.^5^ It can be defined as, “an individual’s inability to control his or her use of the internet, which eventually causes psychological, social, school, and/or work difficulties in a person’s life”.^6^ A previous study done amongst university students revealed that those who did not use Internet excessively had better relationships with administrative staff, better academic grades and increased learning satisfaction as compared to heavy Internet users.^7^

Internet addiction is a relatively new research area.^6^ After extensive literature search, no previous study was found that observed the relationship between excessive internet use and academic performance among the medical students in Pakistan. The current study attempted to investigate the relationship between internet addiction and academic performance of the medical students of Azad Kashmir, Pakistan.

## METHODS

This was a cross-sectional institution-based study carried out in Poonch Medical College in Rawalakot (CMH), Azad Kashmir, Pakistan. The study period was from May 2018 to November 2018. First year MBBS students were excluded from the study as they did not appear in the first professional examination. The total number of second to fifth year medical students in Poonch Medical College was five hundred. The researcher distributed Young’s Internet Addiction Test Questionnaire, purpose of the study, and informed consent letter to all the second to fifth year medical students via email.^8^ Permission to use the questionnaire was secured from the developer Dr. Young. The questionnaire contained twenty 5-points Likert questions to measure internet addiction. Student’s professional exam results were also collected as the academic performance from the academic database of Poonch Medical College (PMC/RKT/14,18 dated March 5^th^, 2013). The research was carried out ethically as stipulated in the Nuremberg code and informed consent was obtained from every participant of the study at enrollment.

### Statistical analysis

Baseline characteristics and responses to the Internet addiction questions were presented as frequencies and percentages. Internet addiction score was calculated by adding all 5-points Likert scale questions for each respondent. Scoring for the Likert scale questions were done as follows: 0 = does not apply, 1 = rarely, 2 = occasionally, 3 = frequently, 4 = often, and 5 = always. Therefore, the highest possible score by a student was 100 and the lowest possible score was zero. Normality tests were done for Internet Addiction scores which revealed non-normal distribution. Internet addiction score was compared with respondents’ age, years in medical college and academic performance by Kruskal Wallis H test. Mann Whitney U test was done to observe the relationship between internet addiction score and gender. Relationship between internet addiction category and academic performance was observed by Spearman Rank Correlation test. Data were presented in tables and figure. The analysis was performed in 95% confidence interval using the Statistical Package for Social Science (SPSS), version 23.0 (IBM, Armonk, NY, USA).

## RESULTS

Out of 500 undergraduate medical students (year 2 to 5), 316 students responded to the study questionnaire and were included in this study (response rate of 63.2%). Majority of the respondents were female 187 (59.2%), and their median age was 22 years. The highest internet addiction score found was 93 by two students and the lowest score was two. The median internet addiction score was 38.

Seventy (22.2%) students always stay online longer than intended. The same number of respondents believe that life without the internet is boring and joyless. When asked about ‘How often do your grades or school work suffers because of the amount of time you spend online?’, 45 (14.2%) replied ‘frequently’, 19 (6.0%) answered ‘often’, and 31 (9.8%) said ‘always’. Responses to all internet addiction questions (Q1 to Q20) were presented in Table-I.

**Table-I:** Responses to the questions of Young’s internet addiction test.

Questions	Does not apply	Rarely	Occasionally	Frequently	Often	Always
1. How often do you find that you stay online longer than you intended?	11 (3.5)	49 (15.5)	54 (17.1)	66 (20.9)	66 (20.9)	70 (22.2)
2. How often do you neglect household chores to spend more time online?	27 (8.5)	66 (20.9)	76 (24.1)	58 (18.4)	49 (15.5)	40 (12.7)
3. How often do you prefer the excitement of the internet to intimacy with your partner?	161 (50.9)	68 (21.5)	30 (9.5)	27 (8.5)	17 (5.4)	13 (4.1)
4. How often do you form new relationships with fellow online users?	101 (32.0)	131 (41.5)	53 (16.8)	17 (5.4)	6 (1.9)	8 (2.5)
5. How often do others in your life complain to you about the amount of time you spend online?	52 (16.5)	92 (29.1)	65 (20.6)	41 (13.0)	40 (12.7)	26 (8.2)
6. How often do your grades or school work suffers because of the amount of time you spend online?	39 (12.3)	110 (34.8)	72 (22.8)	45 (14.2)	19 (6.0)	31 (9.8)
7. How often do you check your email before something else that you need to do?	89 (28.2)	95 (30.1)	47 (14.9)	47 (14.9)	20 (6.3)	18 (5.7)
8. How often do your job performance or productivity suffer because of the internet?	41 (13.0)	87 (27.5)	77 (24.4)	55 (17.4)	34 (10.8)	22 (7.0)
9. How often do you become defensive or secretive when anyone asks you what you do online?	97 (30.7)	84 (26.6)	67 (21.2)	29 (9.2)	18 (5.7)	21 (6.6)
10. How often do you block out disturbing thoughts about your life with soothing thoughts of the internet?	54 (17.1)	81 (25.6)	57 (18.0)	55 (17.4)	39 (12.3)	30 (9.5)
11. How often do you find yourself anticipating when you will go online again?	52 (16.5)	83 (26.3)	61 (19.3)	69 (21.8)	35 (11.1)	16 (5.1)
12. How often do you fear that life without internet would be boring?	29 (9.2)	64 (20.3)	73 (23.1)	38 (12.0)	42 (13.3)	70 (22.2)
13. How often do you snap, yell, or act annoyed if someone bothers you while you are online?	59 (18.7)	106 (33.5)	75 (23.7)	30 (9.5)	21 (6.6)	25 (7.9)
14. How often do you lose sleep due to late-night logins?	25 (7.9)	72 (22.8)	63 (19.9)	38 (12.0)	55 (17.4)	63 (19.9)
15. How often do you feel preoccupied with the internet when offline, or fantasize about being online?	77 (24.4)	82 (25.9)	58 (18.4)	48 (15.2)	27 (8.5)	24 (7.6)
16. How often do you find yourself saying “just a few more minutes” when online?	29 (9.2)	51 (16.1)	53 (16.8)	54 (17.1)	73 (23.1)	56 (17.7)
17. How often do you try to cut down the amount of time you spend online?	33 (10.4)	76 (24.1)	67 (21.2)	59 (18.7)	40 (12.7)	41 (13.0)
18. How often do you try to hide how long you’ve been online?	86 (27.2)	93 (29.4)	52 (16.5)	37 (11.7)	32 (10.1)	16 (5.1)
19. How often do you choose to spend more time online over going out with others?	78 (24.7)	82 (25.9)	65 (20.6)	30 (9.5)	37 (11.7)	24 (7.6)
20. How often do you feel depressed, moody or nervous when you are offline which goes away once you are back online?	75 (23.7)	101 (32.0)	50 (15.8)	42 (13.3)	30 (9.5)	18 (5.7)

**Fig.1:**
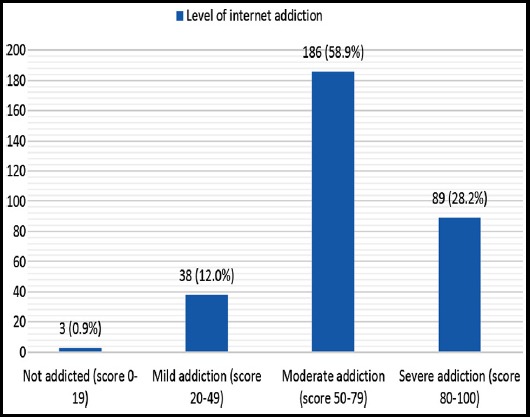
Distribution of all respondents by the level of internet addiction.

As per the internet addiction scores, 89 (28.2%) falls in severe addiction category (score 80-100) whereas only 3 (0.9%) had no internet addiction (Score <20) (Fig.1). Internet addiction score was not statistically significantly different across age and gender. However, IA score was significantly different for the students of different medical years with third year students scoring the highest (mean 43.02 ± 17.35) (p 0.018). Academic performance was also significantly associated with IA score (p <0.001) (Table-II). A mild downhill correlation was found between internet addiction categories (no addiction, mild addiction, moderate addiction, and severe addiction) and academic performance (p 0.013) (Table-III).

**Table-II:** Relationship between Internet addiction score and demographic characteristics, and academic performance.

Characteristics	N (%)	Mean ± SD IA score	Median IA score	p-value
***Age in years***				
19	2 (0.6)	39.00 ± 0.00	39	0.139
20	42 (13.3)	35.43 ± 16.29	36	
21	49 (15.5)	37.86 ± 17.35	37	
22	111 (35.1)	41.50 ± 15.16	42	
23	78 (24.7)	46.21 ± 19.99	43.5	
24	30 (9.5)	34.60 ± 18.46	35	
25	4 (1.3)	8.50 ± 4.04	4	
***Gender***				
Male	129 (40.8)	44.44 ± 18.78	42	0.154
Female	187 (59.2)	37.27 ± 16.63	37	
***Medical years***				
Second	74 (23.4)	40.55 ± 20.21	38	0.018
Third	135 (42.7)	43.02 ± 17.35	41	
Fourth	67 (21.2)	39.79 ± 15.22	42	
Fifth	40 (12.7)	30.70 ± 16.31	31.5	
***Academic performance***				
50-60%	14	33.14 ± 9.19	35.5	<0.001
61-70%	131	44.69 ± 20.20	45	
71-80%	168	37.84 ± 16.31	36.5	
>80%	3	9.33 ± 7.50	5	

**Table-III:** Relationship between internet addiction categories and academic performance.

		Academic performance
Categories of IA	Correlation coefficient	-0.139
	p-value	0.013

## DISCUSSION

A previous Chinese study involving the same Young’s IA questionnaire revealed addiction to instant messaging not only cause a detrimental effect on academic performance but also deteriorates the social and interpersonal relationship.^9^ A similar study done on Malaysian university students also revealed that internet addiction is associated with lower CGPA.^10^ These study findings go in line with our current study findings.

Internet addiction can also cause loneliness, shyness, depression and various other psychological conditions.^11^-^13^ All these factors may make the development and maintenance of the social relationship with the academic supervisors and peers difficult. Moreover, overuse of the internet may expose the students to its dark side like spam, malware, hacking, phishing, invasion of privacy, pornography etc.^14^ Addiction in these dark sides may be career ending.

According to the World Bank data, 22.2% of Pakistanis used internet in the year 2018, which is lower than most of the Asian countries. Internet use will increase over time and due to an increase in overall content and connection speed, there will be more addictive contents. One study suggested internet addiction can be a 21^st^ century epidemic.^15^ No previous Pakistani study was found which revealed in-depth analysis of internet addiction among medical students. However, a previous local study showed excessive internet use caused a negative impact on academic performance of business school students.^16^

### Strength and limitations of the study

According to our knowledge, it is the first study that showed the association between internet addiction and negative academic performance among Pakistani medical students. This study had several limitations e.g. small sample size, single center, and cross-sectional nature of the study, etc. This study does not recommend the generalization of the findings; instead, it recommends further studies in this field.

## CONCLUSION

This study revealed only a handful of medical students were not internet addicted which is very alarming because the internet in Pakistan is still not very cheap and super-fast. Those who were internet addicted showed deteriorated academic performance compared to those who were not internet addicted. Despite several recommendations, internet addiction is still not officially declared as a psychiatric disorder, but it has the potential to be one. Steps should be taken to reduce the misuse of the internet.

### Authors’ Contribution:

**AJ, SKG** conceived, designed and did statistical analysis & editing of manuscript.

**RJ, SG,** did data collection and manuscript writing.

**AJ** takes the responsibility and is accountable for all aspects of the work in ensuring that questions related to the accuracy or integrity of any part of the work are appropriately investigated and resolved.
